# First Complete Genome Sequence of *Tobacco necrosis virus D* Isolated from Soybean and from North America

**DOI:** 10.1128/genomeA.00781-17

**Published:** 2017-08-10

**Authors:** Gustavo A. Díaz-Cruz, Charlotte M. Smith, Kiana F. Wiebe, Bryan J. Cassone

**Affiliations:** Department of Biology, Brandon University, Brandon, Manitoba, Canada

## Abstract

We present the first complete genome sequence of the tombusvirus *Tobacco necrosis virus D* (TNV-D) from North America, obtained from an infected soybean plant. Compared with the three other TNV-D genomes isolated from different geographic regions and host plants, its nucleotide identities were between 83% and 93%.

## GENOME ANNOUNCEMENT

*Tobacco necrosis virus D* (TNV-D) is a single-stranded positive-sense RNA virus in the family *Tombusviridae* (genus *Necrovirus*). First described in *Nicotiana tabacum* (1), the virus can be transmitted experimentally to at least 88 dicotyledonous and monocotyledon species in 37 families (2). While systemic infections are rare and limited to only a few host species, inoculated plants typically show necrotic local lesions (2). TNV-D is not insect vectored; rather, it is naturally transmitted by zoospores of the root-infecting chytrid fungus *Olpidium brassicae*, entering the root at the same time as the fungus (3–5). Although the virus has a broad host range and is likely distributed worldwide, its genome sequence is only available from three isolates: TNV-D infecting French bean (*Phaseolus vulgaris* cv. The Prince) from England (6, 7), TNV-D^H^ infecting tobacco (*Nicotiana clevelandii*) from Hungary (8), and TNV-D^P^ infecting olive trees (*Olea europaea* L.) from Portugal (9). Based on these assemblies, the genome is approximately 3.8 kb in length and contains six protein coding genes.

In August 2016, a leaf sample was collected from a soybean (*Glycine max* [L.] Merr.) plant exhibiting necrotic lesions, growing in a natural field in Manitoba, Canada. Total RNA was extracted using the plant RNA/DNA purification kit (Norgen Biotek) and sent to the Génome Québec Innovation at McGill University for sequencing in 100-bp paired-end fashion on the Illumina HiSeq 2500 platform. After adapter removal and trimming for quality, a total of 50,927,028 reads were obtained. Preprocessed reads were *de novo* assembled using CLC Genomics Workbench 10.0.1 (CLC Bio) and the following parameters: bubble size, 500; similarity fraction, 0.9; length fraction, 0.65; and default settings herein. Functional annotation of the resultant contigs was carried out using BLASTn against the NCBI nonredundant database (E value < 1 × 10^−50^). The TNV-D (Manitoban isolate, TNV-D^MB^) genome length obtained was 3,742 nucleotides (nt) with an average coverage of 812×. The overall genome sequence identities between TNV-D^MB^ and the other TNV-D isolates were 83.4% (TNV-D), 93.3% (TNV-D^H^), and 83.2% (TNV-D^P^). Gene prediction using FgenesV (Softberry, Inc.) and OrfPredictor (10) identified the same six protein coding genes found in the other sequenced isolates in the following order: 22-kDa and 82-kDa genes encoding products required for RNA replication (p22 and p82), three different 7-kDa genes (7_1_, 7a, and 7b) thought to be involved in cell-to-cell movement, and a coat protein (CP) gene. Much like the other isolates, the genome organization is very compact. Only two noncoding nucleotides are present between 7b and CP, and several genes overlap one another (p82 and 7_1_, 3 nt; 7_1_ and 7a, 142 nt; 7a and 7b, 1 nt). This is the first complete nucleotide sequence of a TNV-D isolate from soybean, as well as from North America.

### Accession number(s).

The complete genome sequence of TNV-D^MB^ has been deposited in GenBank under accession number MF158986.
